# Risk Assessment of Intermittent and Continuous Nasogastric Enteral Feeding Methods in Adult Inpatients: A Meta-Analysis

**DOI:** 10.1155/2021/8875002

**Published:** 2021-01-07

**Authors:** Guang Yang, Bojun Zheng, Yi Yu

**Affiliations:** ^1^Department of Critical Care Medicine, The Second Affiliated Hospital of Guangzhou University of Chinese Medicine, Guangzhou 510006, Guangdong, China; ^2^Guangzhou University of Chinese Medicine, Guangzhou 510006, Guangdong, China

## Abstract

Diarrhea and pneumonia are common and serious complications in hospitalized patients requiring nasogastric enteral feeding. Our study aimed to compare the risk of diarrhea and pneumonia between intermittent nasogastric enteral feeding (IEF) and continuous nasogastric enteral feeding (CEF). We systematically searched PubMed, Web of Science, and Cochrane for relevant articles published from August 9, 1992, to September 1, 2019. A total of 637 IEF and CEF patients were included in our meta-analysis. Odds ratios (ORs) with associated 95% confidence intervals (CIs) were calculated to estimate the effects of diarrhea and pneumonia. We showed that hospital patients that required IEF had an increased risk of diarrhea compared with CEF. In the subgroup analyses, similar conclusions were identified in the non-China group and small sample size group (size < 100). However, our results showed no significant differences in the China group or large sample size group (size ≥ 100). Furthermore, our analysis showed that no significant association was observed for the risk of pneumonia between IEF and CEF patients. For inpatients requiring nasogastric enteral feeding, CEF is a better method of enteral nutrition compared with IEF, of which patients experience a significantly increased risk of diarrhea.

## 1. Introduction

Nutritional support is one of the indispensable factors for human survival. At present, enteral nutrition (EN) is the preferred way of nutritional support for severe patients with diminished intestinal function, especially in the Intensive Care Unit (ICU). Enteral provision of caloric intake favorably modulates disease severity [1, 2], immune system function [3], gastrointestinal integrity [4], and mucosal host defenses [5]. EN can be given as intermittent nasogastric enteral feeding (IEF) or continuous nasogastric enteral feeding (CEF) [6, 7]. CEF is thought to be better tolerated by patients with limited absorptive gut surface area or gastrointestinal dysfunction but is associated with more tube clogging and requires the patient to be attached to an infusion pump [7, 8]. IEF is given at standard intervals and is considered to be more physiological with regard to the cephalic phase of digestion and gut homeostasis [9]. However, many researchers believe that IEF could increase the risk of developing gastrointestinal and pulmonary complications when compared to CEF [6, 10].

For patients requiring nasogastric enteral feeding to maintain dysphagia and poor oral intake, their length of hospital stay and quality of life are uncertain [11]. It is essential to prevent complications from nasogastric enteral feeding. Diarrhea is the most common complication in nasogastric enteral feeding, and aspiration of gastric contents often results in a higher risk of pneumonia, which is one of the most common causes of death in tube-fed patients. Therefore, many studies have been conducted in search of ways to best prevent these complications [12–15]. However, there is still controversy with the current research.

Thereby, the aim of the present study was to investigate the risk of diarrhea and pneumonia in IEF and CEF patients from relevant studies to provide a better nutritional feeding method for patients with clinical nasogastric feeding to improve comfort and increase quality of life.

## 2. Materials and Methods

### 2.1. Search Strategy

Systematic literature in electronic literature databases was searched for relevant prospective published studies prior to September 1, 2019. Potentially relevant studies were identified by various combinations of the following terms or keywords: “intermittent nasogastric enteral feeding,” “continuous nasogastric enteral feeding,” “pneumonia,” and/or “diarrhea.” However, we excluded case reports, editorials or letters to the editor, review articles, and non-English studies. The latest study was published between April and June 2011.

### 2.2. Study Selection Criteria

To ensure the reliability of the studies, inclusion criteria were as follows: (1) observational studies with patients with nasogastric tube enteral feeding at least once a day; (2) all studies emphasizing comparison of complications caused by IEF versus CEF; (3) studies providing an accurate number of patients with diarrhea or pneumonia during the investigation; and (4) adult patients ≥18 years old. Included studies satisfied all four inclusion criteria.

Studies were excluded if they met any of the following characteristics: (1) being designed as a review, a case-controlled study, or an animal study; (2) patient age being <18 years old; (3) being not associated with diarrhea or pneumonia; and (4) overlapped or duplicate reports.

### 2.3. Data Extraction

For each of the selected studies, the following items were extracted: authors, year of publication, study regions, mean age, sample size, duration of follow-up, quality scores, and endpoints. The endpoints of the studies included the number of patients that developed diarrhea or pneumonia with IEF and CEF over the study period. We assessed the quality of the studies obtained from the literature search using the Newcastle–Ottawa Scale (NOS). A total score of ≥7 was considered high quality.

### 2.4. Statistical Analysis

Stata version 12.0 (Stata Corporation) was used to perform all statistical analyses. Incidences of diarrhea and pneumonia during IEF and CEF for each study were treated as dichotomous outcomes and expressed as odds ratios (ORs) and accompanying 95% confidence intervals (CIs). We assessed heterogeneity among studies using Cochrane's *Q* and *I*^2^ tests. A fixed-effect model was used if no significant heterogeneity was identified (*I*^2^ = 0.0%) or the random effect model was used.

Considering inconsistent patient characteristics, different methods for IEF, and other confounding factors across studies, we performed sensitivity analysis to evaluate the stability of our results and explored the possible sources of heterogeneity. Potential publication bias was detected by visually inspecting the funnel plots and using the Begg and Egger tests. Because of the small number of studies and patients for other outcomes, we conducted sensitivity analysis and publication bias assessment only for diarrhea and pneumonia in IEF. A two-sided value of *P* < 0.05 was considered statistically significant.

## 3. Results

### 3.1. Literature Search and Included Studies

**Figure 1:**
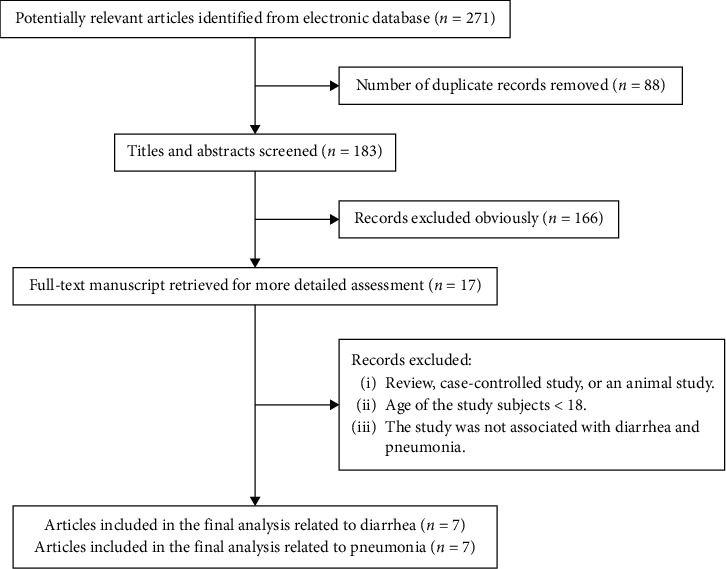
Flow diagram of literature search and study selection.

Detailed instructions of how the studies were obtained are presented in Figure 1. Through the outlined search strategy, we identified 271 papers that were deemed potentially eligible. A total of 183 full-text papers were selected following title and abstract screening, of which 166 were subsequently excluded (Figure 1). We included nine articles [6, 10, 15–21] in the present meta-analysis after the application of the selection criteria. Two studies [17, 20-21] were recognized for inclusion which were only related to diarrhea, and two [16, 19] studies were only related to pneumonia; the remaining five articles [6, 10, 15, 18] included both diarrhea and pneumonia.

The trials included 637 patients with a critical illness, trauma, head-injury, older tube-fed, and mechanical ventilation [6, 10, 15–21]. The characteristics of the individual studies are shown in Table 1. There were two endpoints from the nine studies, including the number of patients with diarrhea [6, 10, 15, 17, 18, 20-21] and the number of patients with pneumonia during IEF and CEF [6, 10, 15, 16, 18–20].

The study size ranged from 18 to 178 patients, and the duration varied between one and 28 days. Nasogastric enteral feeding periods spanned from 1992 to 2011. These trials were all observational studies and were conducted in the USA [6, 10, 15, 16], Hong Kong, China [17, 20], Brazil [18], India [21], and Poznań, Poland [19]. For all the studies, the average age of the patients was >18 years old. Availability of ORs with their 95% CIs was obtained by statistical analysis. According to the NOS, all studies were of great quality and had scores of seven or more.

### 3.2. Diarrhea in IEF and CEF

**Figure 2:**
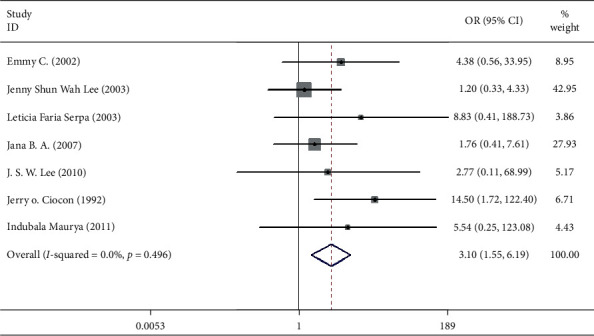
Result of meta-analysis on odds ratio (OR) values for diarrhea. Each square denotes the ORs for each trial comparison with the corresponding 95% confidence intervals (CIs).

**Figure 3:**
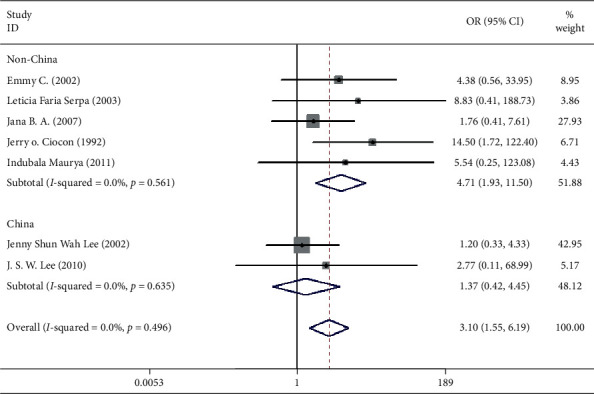
Result of meta-analysis on odds ratio (OR) values for diarrhea. The subgroup is analyzed according to the study region (China and non-China). Each square denotes the ORs for each trial comparison with the corresponding 95% confidence intervals (CIs).

**Figure 4:**
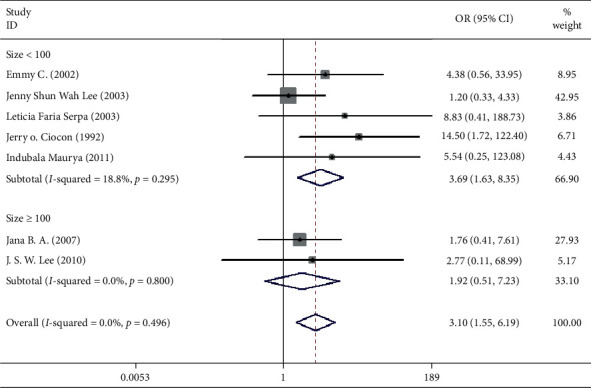
Result of meta-analysis on odds ratio (OR) values for diarrhea. The subgroup is analyzed according to the sample size (size ≥ 100 and size < 100). Each square denotes the ORs for each trial comparison with the corresponding 95% confidence intervals (CIs).

Seven studies including 537 patients documented diarrhea occurrence in patients during IEF and CEF [6, 10, 15, 17, 18, 20, 21]. We observed that IEF increased the risk of diarrhea compared with CEF in hospitals (OR = 3.10, 95% CI = 1.55–1.69, *P*=0.496, random effects, Figure 2). Meanwhile, we performed two subgroup analyses according to the study region (China and non-China; Figure 3) and sample size (≥100 and <100; Figure 4). In comparison with overall research results, similar conclusions were identified in the non-China group (OR = 4.71, 95% CI = 1.93–11.50, *P*=0.561, random effects, Figure 3). However, metaregression showed no significant differences in the China group (OR = 1.37, 95% CI = 0.42–4.55, *P*=0.635, random effects, Figure 3). In the small sample size group (<100), there was an increased risk of diarrhea in IEF patients compared with CEF patients (OR = 3.69, 95% CI = 1.63–8.35, *P* = 0.295, random effects, Figure 4). However, the analysis indicated no significant differences in the large sample size group (≥100; OR = 1.92, 95% CI = 0.51–7.23, *P* = 0.800, random effects, Figure 4). Differences between study region and sample size subgroups were not statistically significant (*I*^2^ = 0.0%, *P* = 0.496; Figure 2).

### 3.3. Sensitivity Analysis for the Risk of Diarrhea

**Figure 5:**
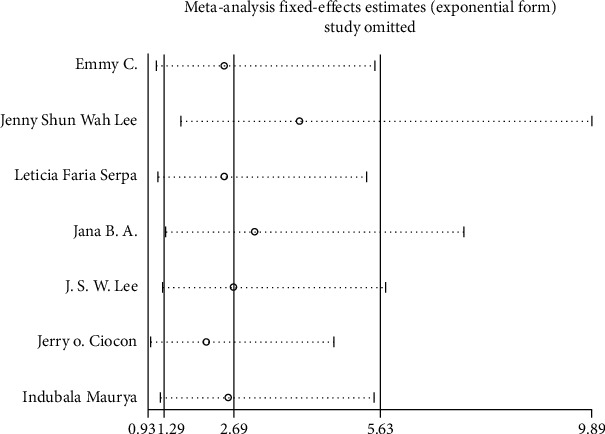
Result of sensitivity analysis for diarrhea. The middle vertical line indicates the combined OR, and the two vertical lines represent the 95% CI = values. Every hollow round indicates the pooled OR when the left study was omitted in a meta-analysis.

**Figure 6:**
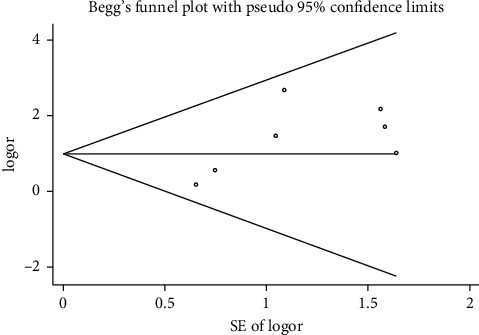
Begg's funnel plot with pseudo 95% confidence limits.

**Figure 7:**
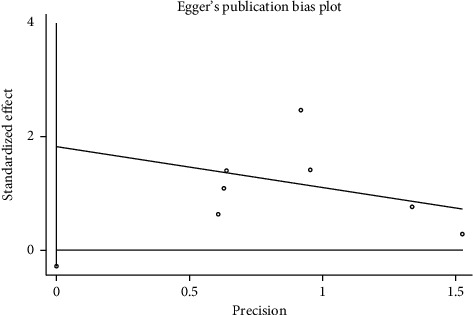
Egger's publication bias plot.

We performed a sensitivity analysis aiming to test the stability of our results. We found no significant differences between the outcomes among all trials, and we did not observe any significant interactions with a range from 0.93 to 5.63 (Figure 5). Furthermore, there was no evident publication bias by funnel plots (Figures 6 and 7).

### 3.4. Pneumonia in IEF and CEF

**Figure 8:**
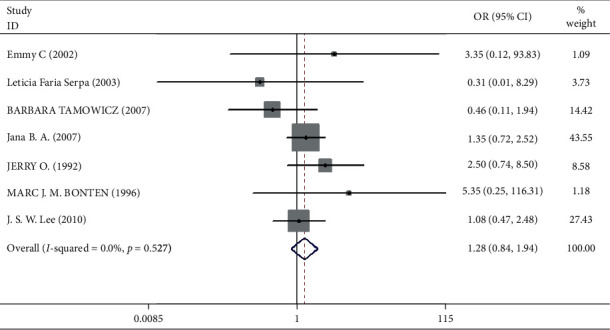
Result of meta-analysis on odds ratio (OR) values for pneumonia. Each square denotes the ORs for each trial comparison with the corresponding 95% confidence intervals (CIs).

**Figure 9:**
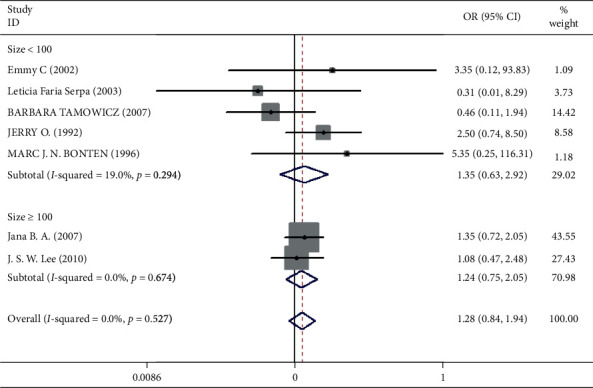
Result of meta-analysis on odds ratio (OR) values for pneumonia. The subgroup is analyzed according to the sample size (size ≥ 100 and size <100). Each square denotes the ORs for each trial comparison with the corresponding 95% confidence intervals (CIs).

**Figure 10:**
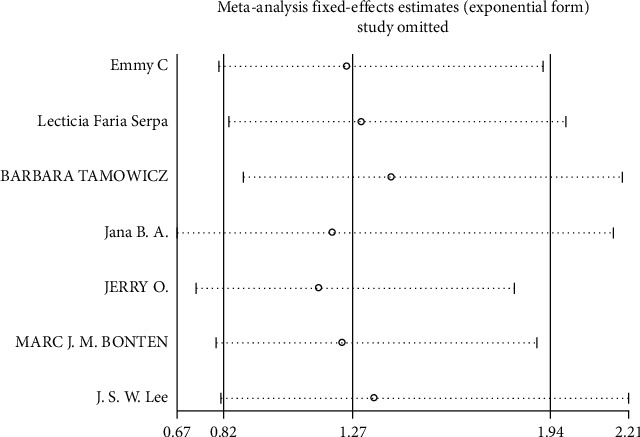
Result of sensitivity analysis for pneumonia. The middle vertical line indicates the combined OR, and the two vertical lines represent the 95% CI = values. Every hollow round indicates the pooled OR when the left study was omitted in a meta-analysis.

**Figure 11:**
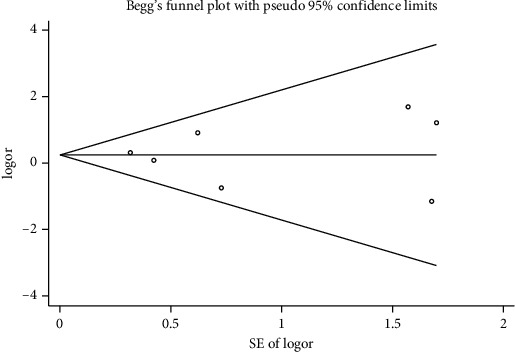
Begg's funnel plot with pseudo 95% confidence limits.

**Figure 12:**
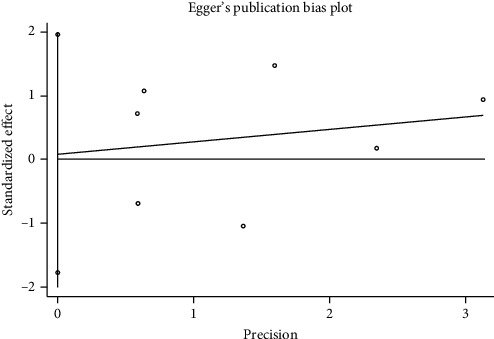
Egger's publication bias plot.

Mortality data were available from seven trials (*n* = 523) [6, 10, 15-16, 18–20]. The combined results showed that no statistically significant association was identified for the risk of pneumonia between IEF and CEF patients (OR = 1.28, 95% CI = 0.84–1.94, *P* = 0.527, random effects, Figure 8). In addition, we performed subgroup analyses according to the sample size of the patient population (large ≥100 and small <100 groups; Figure 9). Similar conclusions were identified between the small sample size group (OR = 1.35, 95% CI = 0.63–2.92, *P* = 0.294, random effects, Figure 9) and large size group (OR = 1.24, 95% CI = 0.75–2.05, *P* = 0.674, random effects, Figure 9). In addition, we performed a sensitivity analysis on these studies and found no significant differences between overall research results, and no notable interactions were observed with a range from 0.67 to 2.11 (Figure 10). Furthermore, no evident publication bias was found using funnel plots (Figures 11 and 12).

**Figure 13:**
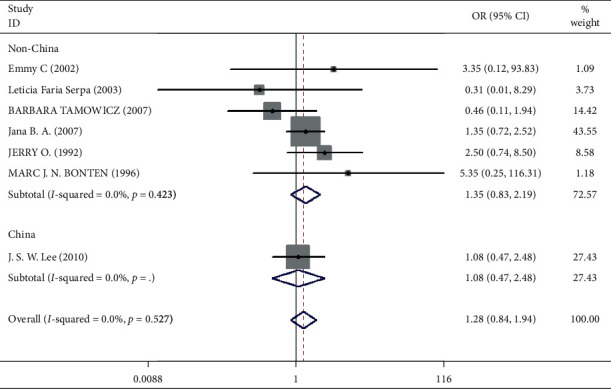
Result of meta-analysis on odds ratio (OR) values for pneumonia. The subgroup is analyzed according to the study region (China and non-China). Each square denotes the ORs for each trial comparison with the corresponding 95% confidence intervals (CIs).

One study [15] was performed in China, and we compared this study with the remaining six studies that were performed in other countries. We observed a similar result as the overall seven [6, 10, 15-16, 18–20] studies (OR = 1.35, 95% CI = 0.83–2.91, *P* = 0.423, random effects, Figure 13). Hence, we suggest that there is no difference in the risk of pneumonia between the two nasogastric enteral feeding methods.

## 4. Discussion

Nutrition plays a significant role in medical institutions. For patients requiring nasogastric enteral feeding, malnutrition can have fatal effects. The American Society of Parenteral Enteral Nutrition (ASPEN) published the 2016 Edition of the Guidelines for the Provision and Evaluation of Nutritional Support Therapy for Adult Critically Ill Patients [22], which suggested nasogastric enteral feeding is preferred for inpatients requiring nutritional support therapy. Enteral nutrition supply and feeding through the stomach is an acceptable enteral nutrition for most patients requiring nasogastric feeding, and it is recommended that the intestinal nutrition tolerance should be monitored daily to avoid inappropriate termination of enteral nutrition. At present, there are a few studies on feeding methods in clinical practice. Hospitals mainly provide enteral nutrition in two ways, including IEF and CEF. Our meta-analysis focused on determining which method is more scientific and humane.

According to various studies, CEF maintains the relative sterility of the nutrient solution, stability of food osmotic pressure, avoids contamination, and reduces many related complications [6, 10]. Additionally, IEF has a certain extent of security and rationality [6, 10]. Current studies have already indicated that there is no correlation between gastric contents and reflux aspiration when the residual amount in the stomach is <500 ml [23–25]. Therefore, the IEF method with an intermittent nutrient solution of approximately 500 ml is safe and suitable for physiological and clinical needs. However, many researchers believe that IEF could increase the risk of developing gastrointestinal and pulmonary complications [12–15]. To determine the risk factors between IEF and CEF, we conducted a meta-analysis that included nine studies [6, 10, 15-21] (seven studies related to diarrhea and six studies that included pneumonia).

The present study showed that patients had a higher risk of developing diarrhea using the IEF method [6, 10, 15, 17, 18, 20, 21]. Because our meta-analysis results should be interpreted cautiously, we carried out a subgroup analysis. We found that most studies (non-China and small sample size groups) resulted in similar conclusions when compared with the overall study results. However, it is important to note that there was no significant difference in the China group and the large sample size group (≥100). Although sensitivity and heterogeneity analysis showed that the results for risk of developing diarrhea were not dominated by individual studies, our meta-analysis results remain interesting.

There is no evidence that a significant association exists between the risk of pneumonia in IEF or CEF patients. There are several possible explanations for why the risk of pneumonia between IEF and CEF is not significant. Tracheobronchial aspiration of gastric contents is a recognized risk factor for pneumonia in the critically ill and the elderly. However, the rate of gastroesophageal regurgitation was not different between IEF and CEF in a previous study [26]. In addition, Simme et al. showed that the stomach is rarely a reservoir of pathogens causing pneumonia; therefore, IEF with a high gastrointestinal tract complication rate does not indicate a high incidence of pneumonia [27]. A previous study has demonstrated that CEF is a risk factor for stomach colonization with potentially pathogenic microorganisms due to an increase in stomach pH instead of IEF [28]. However, the exact incidence of pneumonia is difficult to interpret from our selected articles as these were short-duration studies [6, 10, 15-16, 18-19], which is one of the limitations in our meta-analysis.

The current systematic review and meta-analysis article has several limitations. One potential limitation is the large time span in the included studies. Excessive differences in research years ranging from 1992 to 2011 could cause deviations in results because of the development of nursing technology, treatment technology, and materials used. In addition, the follow-up duration and the difference in the study population may lead to heterogeneity. However, we were not able to detect a major source of heterogeneity in the subgroup and sensitivity analyses.

## 5. Conclusion

In conclusion, IEF patients experienced a significantly increased risk of diarrhea and no evidence of an increased risk of pneumonia compared with CEF patients. Therefore, CEF is a better method of nasogastric enteral feeding for hospital inpatients.

## Figures and Tables

**Table 1:** Main characteristics of the included studies.

Study (year)	Country	Sample	Mean age (years)	Diarrhea in IEF	Diarrhea in CEF	Pneumonia in IEF	Pneumonia in CEF	Follow-up (days)	Quality (Newcastle–Ottawa Scale)
Jerry o. Ciocon (1992)	USA	60	72	29	20	10	5	7	8
Marc J. M (only pneumonia) (1996)	USA	60	66.5			2	0	3	7
Emmy C. (2002)	USA	18	36.6	5	2	1	0	7	7
Jenny Shun Wah Lee (only diarrhea) (2003)	Hong Kong, China	74	82.05	6	5			5	8
Letícia Faria Serpa (2003)	Brazil	28	67.25	3	0	0	1	3	8
Jana B. A. (2007)	USA	139	46.52	5	3	38	33	7	7
Barbara Tamowicz (only pneumonia) (2007)	Poznań, Poland	40	18–75			4	7	6	8
J. S. W. Lee (only diarrhea) (2010)	Hong Kong, China	178	Over 60	1	0	14	12	28	8
Indubala Maurya (only diarrhea) (2011)	India	40	40.45	2	0			1	8

IEF: intermittent nasogastric enteral feeding; CEF: continuous nasogastric enteral feeding.
